# Editorial: Understanding cross-cultural differences through cognition and perception analysis: integrating neuroscience and cultural psychology, volume II

**DOI:** 10.3389/fpsyg.2024.1416853

**Published:** 2024-05-24

**Authors:** Tachia Chin, Chien-Liang Lin, Francesco Caputo, Fengpei Hu

**Affiliations:** ^1^College of Business, Honghe University, Mengzi, China; ^2^School of Management, Zhejiang University of Technology, Hangzhou, China; ^3^Department of Information Management, Ming Chuan University, Taipei, Taiwan; ^4^Department of Economics, Management, and Institutions, University of Naplesn Federico II, Naples, Italy

**Keywords:** cross-cultural differences, cognitive difference, perception analysis, neuroscience, cultural psychology

## Introduction

The COVID-19 crisis has examined the existence of considerable cross-cultural variances in human cognition and perception at both individual and social levels, as described by the well-known individualism-collectivism and holistic-analytic mental paradigms. More specifically, during the pandemic, people all around the globe were facing serious psychosocial challenges elicited by enforced social distancing, mandate quarantine and remote working. However, as reflected by the hot topic whether citizens should wear masks in the public, evidence indicates that culturally-diverse people might perceive various levels of tensions between personal liberties and societal constraints, thus triggering distinct catastrophic feelings toward the same emergency or disaster (Cupples and Glynn, 2014; Chin et al., 2022b).

Notwithstanding it is recognized that people with different cultural beliefs vary in their tolerance to environmental stress as well as in their accepted standards of social norms (Chin et al., 2021), hitherto the currently-fragmented results and limited empirical findings have still left a lot of controversies and puzzles unsolved. Further, many classical studies concerning relevant issues place a major emphasis on explaining causal relevance and attribution between culture and human's mental activities (Choi, 2003; Redding, 2017), rather than exploring the underlying cerebrum mechanisms driving the development of cognition. In response, the main motivation behind this Research Topic (RT) is to fill the lacuna by demonstrating cultural variations through a more modern, more neuroscientific cognition and perception analysis.

According to the literature (Hofstede et al., 2010; Chin et al., 2022a; Han, 2022), the discipline of cultural psychology focuses on elucidating how cultural factors involving mental programming influence human cognition and perception, while the domain of neuroscience explains the forming process of human cognition and perception through exploring robust activators of the hypothalamic-pituitary-adrenal (HPA) axis of human brains, the neuroendocrine control system of human responses to stressors. Whereas, we believe that no single discipline, methodology or instrument can perfectly address the identified gap noted preciously, it seems particularly meaningful to advance to adopt an unconventional, integrative view that links the more objective, scientific approach of neuroscience to profound cultural meanings for making better sense of cross-cultural variances.

Taking together the arguments above, the aim of this RT is to call for interdisciplinary studies at the intersection of cultural psychology and neuroscience that may offer new insights, unorthodox theoretical frameworks or novel methodologies for achieving a deeper, more comprehensive understanding of cross-cultural difference in the post pandemic world riddled with political uncertainty and inter-cultural conflicts. Moreover, the central term of “culture” is defined in a broader way, so as to encourage authors to consider all levels of analysis about cultural differences.

Fortunately, we are very pleased to claim that in total, 49 submissions were received, of which 16 fascinating articles by 60 authors went through rigorous peer-review processes and have been published. To more clearly address the unique value of our SI, below we further classify the 16 articles into three categories based on the methodology used, the main findings discussed and the key implications provided.

## Conceptualizing novel notions converging on cultures and neuroscience

The first part contains three published articles that propose novel terms, or identify, decode and interpret existing notions with unconventional thoughts at the congruence of cultural psychology and neuroscience. Their rationales are mostly built upon not only knowledge from the two domains but also knowledge from other domains, which highlights the complex interdisciplinary nature of our topic. Despite economic integration among countries has long been an important theorizing logic for international business, recent research has shed light on the crucial role of cultural differences in hindering international communication, negotiations and transactions for doing business (Chin et al., 2023).

Echoing this stream of research, Agbanyo and Wang adopts a neuroeconomic perspective that takes into consideration the neural mechanisms of human brains in economic decision-making (Camerer et al., 2005) to conceptualize the sense-making process for international trades. More specifically, they first identified several cultural types based on the diversities in human cognition (e.g., monochronic vs. polychronic cultures; formal vs. informal cultures) and then formulated some patterns for doing international trades between specific nations. For instance, the culture of United Kingdom is deemed to be monochronic, moderately formal and transaction-oriented while the culture of Saudi Arabia is polychronic, formal and relationship-oriented. Given the infancy of using a neuroeconomic perspective to frame cognitive diversity, this article is still far from mature but it indeed provides new insights into understanding cross-cultural differences.

The second paper written by Yang et al. clarifies the notions of “the tragedy of the commons” and “the tragedy of the anticommons” from an unorthodox angle of cognition and perception. The former term occurs when individuals have access to a shared resource and act in their own interest at the expense of other individuals, which may lead to overconsumption, underinvestment, and depletion of resources. The latter occurs when a resource has many owners, of whom all are able to exclude others from using it, which may result in the under-utilization of that resource. It is recognized that the two notions that have been widely applied for understanding an agent's decision making based on perceived value can be influenced by socio-psychological factors. This article thus further points out the need to articulate the paradoxes between the two notions within a context with multiple cultural values.

The third paper by Huang and Agbanyo proposes a novel term of multicultural neurolinguistics involving the integration of psycholinguistics and neurolinguistics to characterize how and why cross-cultural variances in cognition may trigger various neurocognitive mechanisms in the process of multilingual translation between source language (SL) and target language (TL). Using a contest of tonal languages (Chinese) and atonal language (English) multilingual exchange as an example, it is discovered that despite the abundance of translation theories, the absolute clarity on the complex cross-cultural dimension of languages remains scare. In response, this research thus incorporates the view of neuroscience into blending cross-cultural diversity and neurolinguistics as a one-in-all translation approach of “multicultural neurolinguistics” between an SL and a given TL to frame more varieties of translation barriers caused by cross-cultural misunderstanding.

## Empirical studies on cultural cognition and behavioral outcomes

The second part include six published articles, which, from an integrative view of psychology and neuroscience, adopt a variety of empirical methods to address the characteristics of different levels of cultures with idiosyncratic values, beliefs, sagas and languages and their mechanisms on human cognition, perception, and behavior in a given context. Five of the selected six papers use China as their research setting while one is a comparative analysis on social anxiety across nations. This Research Topic of articles enriches the literature by addressing the unique cognitive causes and consequences of different cultures manifested by culturally-distinct groups such as family culture, sporty culture to innovation culture in their respective fields (e.g., finance, sports and tourist industries).

There are two papers focusing on demonstrating the uniqueness of Chinese family culture and its influences on individual and family cognition and behavior. Guo and Liu examine how children's cognitive development and socialization processes are shaped by their family cultural environment. Picture drawing reflects individuals' perception information, thus being able to more easily characterize children's knowing of the world and their emotional expression. Hence, in light of the rules of the drawing elicitation interviews methodology, this research conducts the technique of children's projective drawing as a knowledge base, supplemented by brief interviews to generate results. Based on 321 children's drawings about the tourism elements, the authors summarize main cognitive contents of children's parent-child tourism experiences engrained with their respective family cultures. Moreover, it is also discovered that when children grow up, their cognitive focus seem to shift from the macro level to the micro level, as well as from concrete to abstract; moreover, children pay more attention to bright and vibrant colors during trips. Their findings provide valuable and feasible implications to the burgeoning market of parent-child tourism. Another paper is written by Li Z. et al.. Their research adopts an unconventional cultural angle to interpret the salient discrepancies in household financial decisions between Chinese urban and rural families. Referring to the theoretical framing of Hofstede's cultural dimensions, this article develops hypotheses about how the major cognitive differences between urban and rural families affect their financial asset allocation. Using the data from China Family Panel Studie, results show that family cultural differences are significantly related to their household financial decisions and that, for rural families with high collectivism and uncertainty avoidance, the mediating mechanism of knowledge acquisition on the above-mentioned association is more prominent. To a certain extent, this research is instrumental to China's practitioners and policy makers to reduce the crucial wealth gap between urban and rural families and thereby realize common prosperity.

There are two articles linking group culture to self-reported anxiety. Built upon the social construal theory, Krieg and Xu conduct a comparative study to investigate cross-cultural cognitive differences between two ethnical groups (Japanese and European Americans) in self-reported social anxiety, Using the samples of college students, results show that after selective attention is experimentally manipulated, they found significant cross-cultural differences in self-reported social anxiety and anxious behavior in a speech task. Their findings confirm that social anxiety is culture-bound. However, in contrast, using the Sport Anxiety Scale-2 developed in the West, Li S. et al. evaluate the psychometric properties of Chinese adolescent athletes in taking the National Sports College Entrance Examination. Although their findings examine the cross-cultural applicability of a cognitive measurement on anxiety, this research implies that culture may neither a cause nor a consequence under some circumstances.

In terms of the final two papers in this category, Zhao et al. characterize the links between the performing of a couple of interaction behaviors at group level (i.e., idea facilitation, team spirit facilitation, idea inhabitation, team spirit inhabitation, and neutral interaction behaviors) and team cultures (i.e., low or high innovation culture). Referring to the input-process-output framework, this research adopts a novel experiment methodology to collect data. After coding and analyzing 1,754 behaviors, the authors suggest that innovation cultures seem to show no effect on interaction behaviors among team members, thus providing some interesting implications to the practice. Wang et al. undertake an electroencephalography (EEG) experiment to explore the mechanisms of country-of-origin (COO) stereotypes-brand positioning congruence on consumer behavior. Results indicate that consumers display a higher purchase intention in the congruence condition over incongruence condition. Moreover, from the angle of brain science, the neural data provide evidence supporting the discovered behavioral outcomes because the frontal theta-band oscillation that alludes to cognitive conflicts was found in the COO stereotype-brand positioning incongruence condition. Overall, their findings are beneficial to the formulation of brand positioning strategy at the intersection of culture and neuroscience.

## Digital culture shaping human cognition, perception, and behavior

The third category includes seven published articles that focus on discussing how, by what means and with what possible effects advanced digital technologies and social media use affect human's brain function, cognition and behavior. Referring to the definition of culture (Hofstede et al., 2010), digital culture can be seen as values and practices that developed from the widespread use of digital technologies. Along with the accelerated speed of digitalization, an increasing number of research in psychology, neuroscience and other inter-disciplinary domains have investigated the effects of digital media use on brain function and mental health. Echoing this trend, this Research Topic offers a fundamental rationale and interpretation for understanding different digital cultures and their role in shaping mental programming and behavioral intention of humans.

The first part contains two published papers focusing on linking digital culture to charity website-related issues. Considering the critical importance of peer influence on donation decision-making in social media-based donation platforms, Ye et al. first examine that the number of donated peers has a positive impact on donation behavior and then extract event-related potential (ERP) from electroencephalographic data to unveil the underlying neural reactions regarding perceived emotional rewards and potential risks. Zhang et al. based on the Stimulus-Organism-Response theoretical frame and a context of medical crowdfunding explore the role of perceived trust in affecting donation behavior online as well as the mediating effect of social presence and perceived differences in trust. Using a questionnaire survey, their results support their hypotheses. Overall, the two articles provide novel, valuable findings that enable online charity platforms get better fundraising through a better understanding of their users' psychological process for making a donation.

The second part includes two articles that adopt modern machine learning (ML) techniques to forecast cognitive functions of human brain. Li W. et al. propose a predictive model of cognitive impairment in the elderly based on a novel machine learning (ML) algorithm. More specifically, the authors extract demographics, lifestyle, nutrition, physical inflammation, and blood lipids as key parameters to analyze large population-representative sample of older adults, whereby a well-performed risk forecasting model is constructed for predicting the occurrence and development of cognitive impairment of older adults. Su et al. apply a variety of ML techniques, ranging from random forest (RF), support vector machine (SVM), logistic regression (LR), to neural network (NN) algorithms, to predict learning outcomes of students. Their findings indicate that the NN algorithm seems to be the best ML tool for forecasting students' performance under environment of web-based interactive learning. In sum, the selected two articles examine that machine learning as a subfield of artificial intelligence (AI) indeed provides new opportunities and challenges to operationalize mass data as well as to process previously untapped sources of data.

In terms of the other three papers in this Research Topic, they may not be able to directly enhance or deepen our understanding of cross-cultural differences in human cognition and perception, but rather provide indirect, subtle insights into relevant issues. Fu et al. form a new BCG matrix, aiming to gain a better understanding of the link between customer perception and online shopping modes in the e-commerce platform. Liao et al. probe into the relationship of electronic communication during leisure time and withdrawal behavior in the Chinese context. Their findings show a double-edged (U-shaped) association between the two above-mentioned variables that reflects cultural idiosyncrasies, whereby the authors further build a Chinese culturally-specific analytical framework of employee-cognition-behavior continuum. Xu et al. employ Python to conduct text mining of firms' annual reports, and thereby analyze the complex mechanisms of digitalization on ambidextrous innovation at the firm level through a cognitive analysis.

## Conclusion

Overall, the 16 selected articles of our RT categorized into three collections encompass a wide range of subjects, themes, and disciplines, while the sophisticated methodologies used and the innovative theoretical models built represent a novel synthesis of cultural psychology and neuroscience. The first category is entitled “Conceptualizing novel notions converging on cultures and neuroscience”, in which new ideas at the congruence of cultural psychology and neuroscience are identified, conceptualized or refined; the second is “Empirical studies on cultural cognition and behavioral outcomes”, which could shed light on describing, deciphering, and rationalizing new, unfamiliar phenomenon from an unconventional angle; the third is “Digital culture shaping human cognition, perception and behavior”, which elucidates the complex mechanisms of state-of-the-art digital technologies on human cognition and perception. Notwithstanding the majority of the published articles come from China, we are still glad to see many international cooperation in authorship and affiliations- Which to a certain extent echoes the spirit of our RI to promote cross-cultural understanding through the conduct of collaborative research beyond national boundaries.

According to the recent claims of United Nations (UN), the world has ushered a “new era of conflict and violence” (United Nations, 2023). More explicitly, we are facing a highly dynamic and uncertain world where there exist unresolved regional tensions, violation of international law, illicit economic gain, yawning gap between rich and poor, and the scarcity of resources exacerbated by climate change. These crucial inter-cultural challenges may drive a variety of sensory and mental changes, alluding to an urgent imperative for academics, practitioners and policy makers to gain a more profound, more comprehensive understanding of cross-cultural differences in human's cognition and perception; it is indeed a critical time for us to enhance care, passion and compassion for not only the self, but for others, the society and the environment.

## Author contributions

TC: Conceptualization, Writing – original draft. C-LL: Formal analysis, Writing – original draft. FC: Project administration, Writing – review & editing. FH: Project administration, Writing – review & editing.
